# The Predicted Implications of Paederin Exposure to the Eye: A Paederus Outbreak During the Sudan War in 2025

**DOI:** 10.7759/cureus.92348

**Published:** 2025-09-15

**Authors:** Khalil Ali Ibraheim, Hassan Yousif Adam Regal, Abdalkhaleg Adam Mohammedani, Hussain G Ahmed

**Affiliations:** 1 Surgery, Faculty of Medicine, University of Kordofan, El-Obeid, SDN; 2 Parasitology, Faculty of Medical Laboratory Sciences, University of Kordofan, El-Obeid, SDN; 3 Preventive Medicine, Vector Control Unit, Ministry of Health, North Kordofan State, El-Obeid, SDN; 4 Pathology, Prof Medical Research Consultancy Center, El-Obeid, SDN; 5 Histopathology and Cytology, Faculty of Medical Laboratory Sciences, University of Khartoum, Khartoum, SDN

**Keywords:** eye, ocular dermatitis, paederin, paederus, paederus dermatitis, sudan

## Abstract

Background: The Sudanese armed conflict has severely harmed the health system, causing infectious disease outbreaks. Paederus ocular dermatitis, induced by the toxins (paederin) of *Paederus* beetles, exhibits a clinical presentation that closely resembles several other conditions. This study aims to assess the potential effects of paederin on the ocular region during the *Paederus* outbreak in the Sudan War of 2025.

Methods: This is a hospital-based cross-sectional study. All patients with paederin contact with the eye who presented to the Ophthalmology Clinic of the first author in El-Obeid City, North Kordofan State, Sudan, between 5 July and 5 August 2025 were enrolled for the study. The patients selected had no previous eye disease (specifically infectious conjunctivitis, corneal ulcers, and chronic dermatitis). Out of 250 patients who presented to the clinic with paederin eye contact, only 100 were included in the study. Each of these patients was assessed for ocular clinical symptoms at the initial presentation using the available instruments and devices in the clinic.

Results: Out of the 100 included patients, periorbital dermatitis was found in 80% of cases, followed by itching in 85%, pain in 80%, hyperpigmented skin around the eye in 70%, lid ulcer in 38%, redness in 36%, and lid swelling in 28%. Only 2% of women experienced conjunctivitis, while 8% developed vesicles. The reported number might be much lower than the true number of casualties due to the exclusion criteria.

Conclusion: The massive outbreak of *Paederus* may be associated with the ramifications of the current armed war in Sudan. The ocular clinical manifestation is analogous to that seen in other outbreaks in different areas.

## Introduction

Paederin is an inhibitor of protein synthesis that obstructs cell division, resulting in vesiculation and necrotic degeneration of the epidermis. The resulting damage promotes subsequent infection, particularly under harsh settings [1]. Paederin is a powerful poison present in the hemolymph of *Paederus* beetles, a specific category of rove beetles. It is a vesicant, which induces blistering and irritation upon skin contact, resulting in *Paederus* dermatitis. It is globally dispersed; however, it is more prevalent in rainy seasons and warm climates. The clinical signs consist of vesicular-pustular lesions on erythematous skin. Treatment involves cleansing with soap and water to counteract the toxin's effects and the application of topical steroids in brief intervals [2].

Paederin is a toxic compound identified in rove beetles belonging to the *Paederus *and *Paederidus* species, characterized as a linear polyketide. The compound includes a six-membered tetrahydropyran ring and incorporates several essential structural elements, such as an N-acyl aminal bridge and two dihydropyran rings. Paederin includes an N-((6-(2,3-dimethoxypropyl)tetrahydro-4-hydroxy-5,5-dimethyl-2H-pyran-2-yl)methoxymethyl)tetrahydro-2-methoxy-5,6-dimethyl-4-methylene-D-manno-Nonitol moiety. Several additional compounds, including diaphorin, psymberin, nosperin, and onnamides, are classified within the paederin family [3].

The most common complaint caused by these toxins is burning, followed by pain and blisters in 80.0% and 20.0% of patients, respectively. Lesion duration averaged 4.2 days, with a standard deviation of 1.3 days. Linear lesions dominated the clinical pattern at 40.0%. Erythematous lesions with a central gray area were 30.0%, kissing lesions 20.0%, and burnt lesions 10.0%. Facial lesions dominated this investigation at 40.0%. Leg lesions made up 20.0%, followed by axilla, chest, arm, and back lesions at 10.0% each [4,5].

Ocular symptoms are usually secondary to transfer via the fingers with paederin from elsewhere on the skin, usually exposed skin of the face, neck, or arms. Paederin causes unilateral periorbital dermatitis with or without keratoconjunctivitis. Mild periorbital dermatitis causes immediate discomfort and erythema that lasts 24 to 48 hours. Moderate to severe toxin exposure causes severe neuralgia and erythema 24 hours after contact and a vesicular stage 48 hours later [6].

*Paederus* is frequently documented in East Africa, especially in Tanzania, Kenya, and South Sudan [7-9], yet it is relatively rare in Sudan. Therefore, this study aimed to assess the predictable consequences of paederin contact with the eye during an outbreak of *Paederus* that occurred during the Sudan War in 2025.

## Materials and methods

Study design

This hospital-based cross-sectional study assessed the effects of eye exposure to paederin toxins during an outbreak of Paederus that occurred during the Sudan War in 2025. It was conducted at the Ophthalmology Clinic of the first author in El-Obeid City, North Kordofan State, Sudan, from July 5 to August 5, 2025. This study is reported per the Strengthening the Reporting of Observational Studies in Epidemiology (STROBE) guidelines [10] and was approved by the Human Research Ethics Committee of Prof Medical Research Consultancy Center (EL-Obeid, NK, Sudan) (approval no. HREC 0020/MRCC.7/25). 

Inclusion and exclusion criteria

Of the 250 patients experiencing paederin eye contact who presented to the clinic within the designated timeframe, only 100 were included. All patients exhibiting ocular dermatitis and consenting to participate met the eligibility criteria, irrespective of any demographic characteristics. The criteria for exclusion encompassed infectious conjunctivitis and corneal ulcers, as well as pediatric cases of spring catarrh or other forms of chronic dermatitis.

Sampling

The sample size included a full coverage sample within one month. The consultant ophthalmologist conducted comprehensive investigations, and the anticipated findings were documented. Each patient with ocular dermatitis was selected, and a complete ophthalmic history was taken, including symptoms and the presence of dermatitis in other parts of the body. Complete ophthalmic examinations were performed using a slit lamp and torch.

Data collection and analysis

The most encountered clinical manifestations were periorbital dermatitis, itching, pain, skin hyperpigmentation, lid ulcer, redness, lid swelling, discharge, keratitis, conjunctivitis, and vesicles. The data for this study were first organized into a data sheet before being imported into SPSS Statistics (IBM Corp., Armonk, NY, USA). Frequencies, percentages, means, and cross-tabulations were collected.

## Results

**Figure 1:**
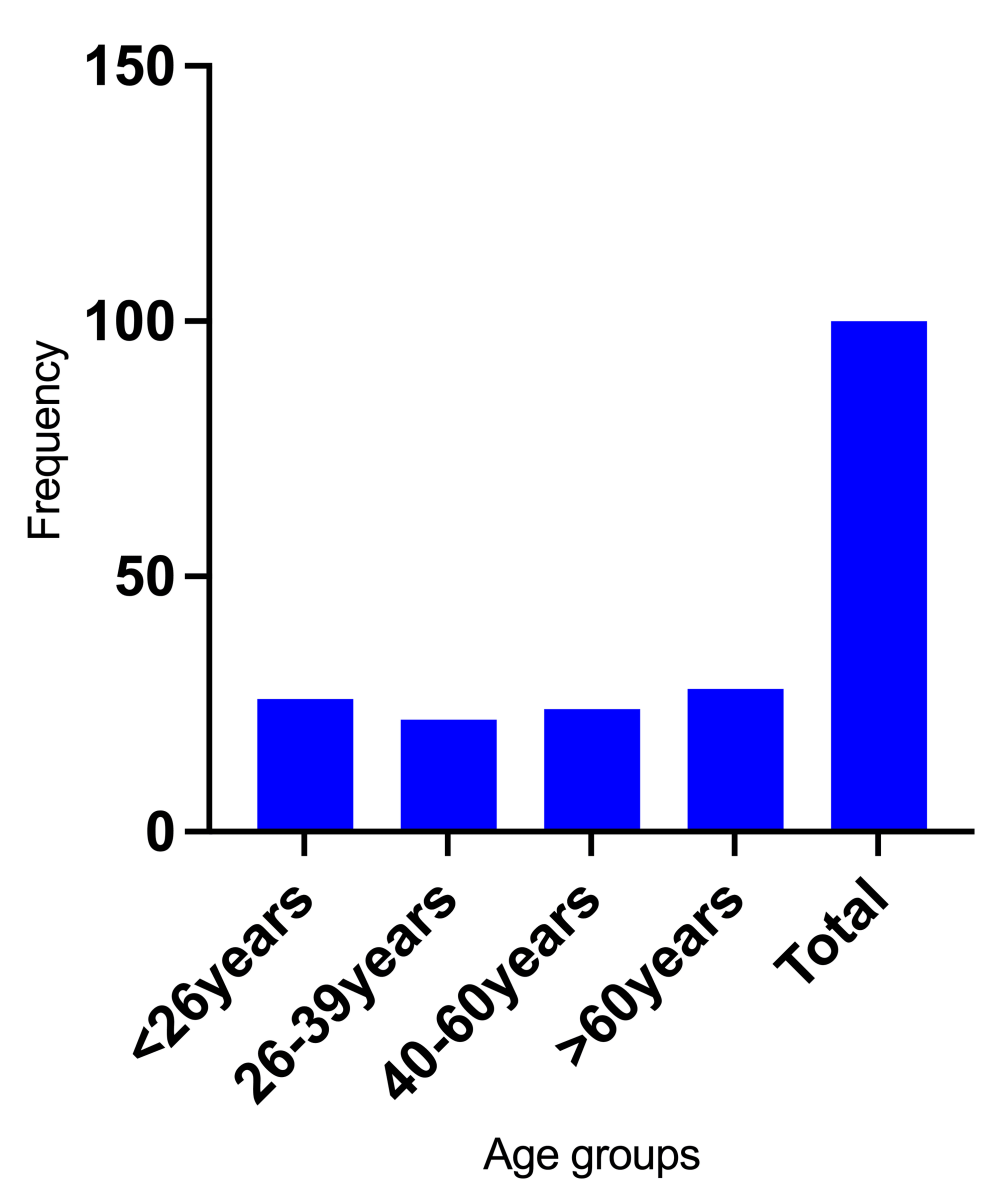
Description of the study subjects by age

Among the 100 patients with ocular manifestations of paederus dermatitis, 60 were male (60%) and 40 were female (40%). The ages ranged from one to 80 years, with the most affected group being those over 60 years (28%), followed by individuals under 26 years (26%), those aged 40 to 60 years (24%), and finally, those aged 26 to 39 years (22%), as illustrated in Figure 1.

**Figure 2:**
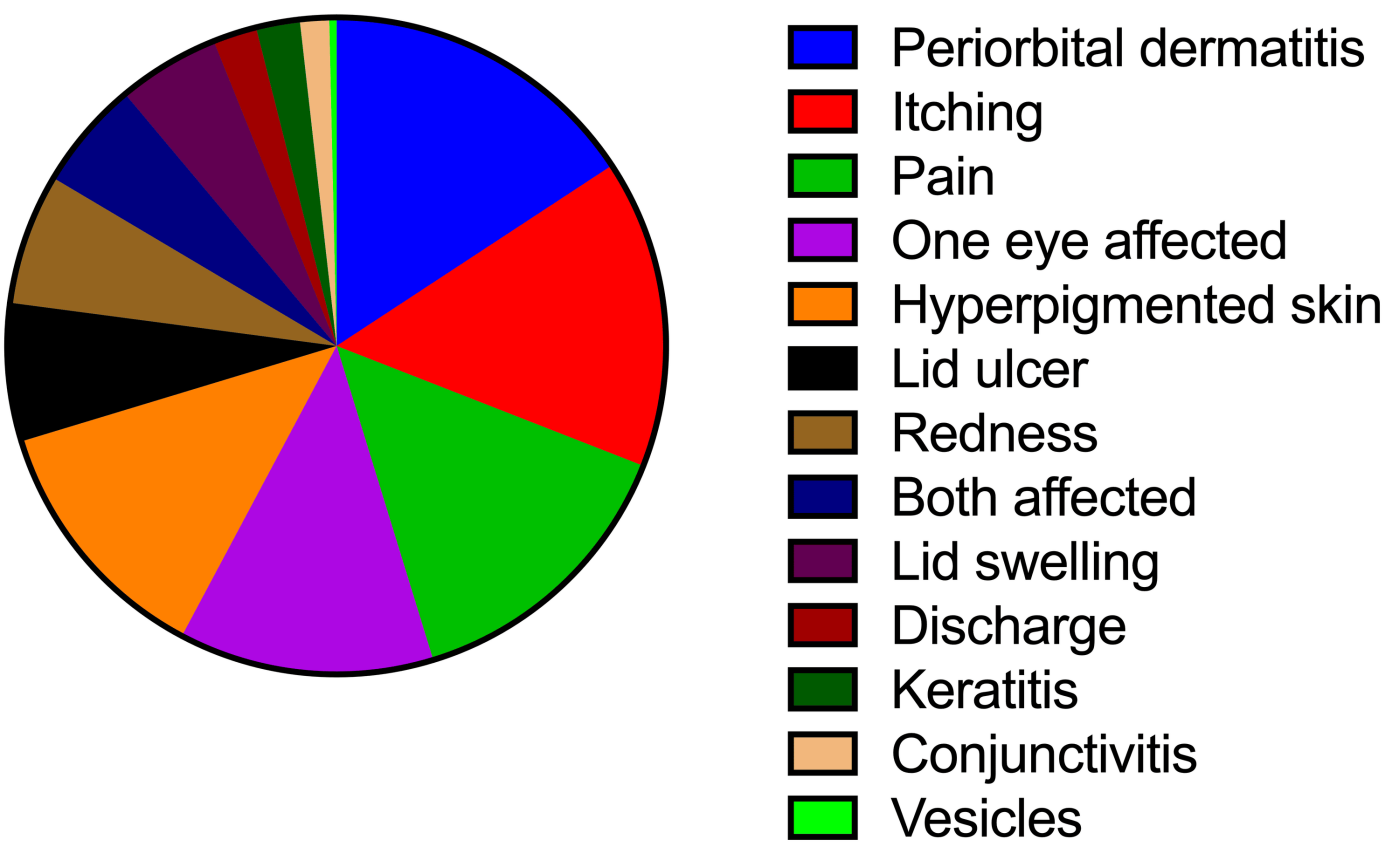
Description of the patients by proportions of clinical manifestations

Clinical signs of this outbreak varied. The most common manifestation in this study was periorbital dermatitis (80%), followed by itching (85%) and pain (80%). Most cases (70%) included one eye, whereas the rest involved both. About 70% of cases had hyperpigmented skin around the eye, 38% had lid ulcers, 36% had redness, 28% had edema, 28% had discharge, and 12% had keratitis. Figure 2 shows that 8% of cases had conjunctivitis and 2% had vesicles, both of which were only found in females.

**Figure 3:**
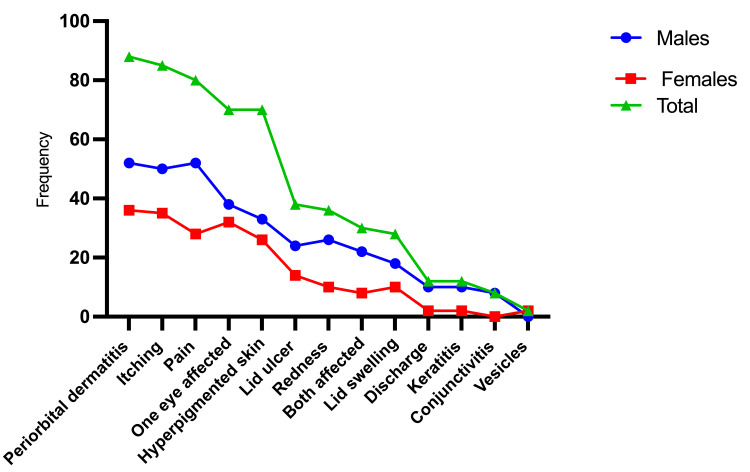
Distribution of the patients by sex and clinical manifestations

Gender distribution of this clinical symptom. Males had 50 cases (56%) with periorbital dermatitis, whereas females had 38 (44%). Itching occurred in 85 cases: 50 males (59%) and 35 females. Hyperpigmented skin was found in 44 (63%) males and 26 (27%) females. One eye was affected in 38 males (54%) and 32 females (46%), while both eyes were affected in 22 (73%) males and eight females (27%). Lid ulcer was present in 24 males (63%) and 14 females (37%). Redness was seen in 26 males (72%) and 10 females (28%). Lid swelling was observed in 18 males (64%) and 10 females (36%), as indicated in Table 1 and Figure 3.

**Table 1:** Patients categorised by sex and clinical manifestations

Clinical manifestations	Males	Females	Total
Periorbital dermatitis	52	36	88
Itching	50	35	85
Pain	52	28	80
One eye affected	38	32	70
Hyperpigmented skin	33	26	70
Lid ulcer	24	14	38
Redness	26	10	36
Both affected	22	8	30
Lid swelling	18	10	28
Discharge	10	2	12
Keratitis	10	2	12
Conjunctivitis	8	0	8
Vesicles	0	2	2

**Figure 4:**
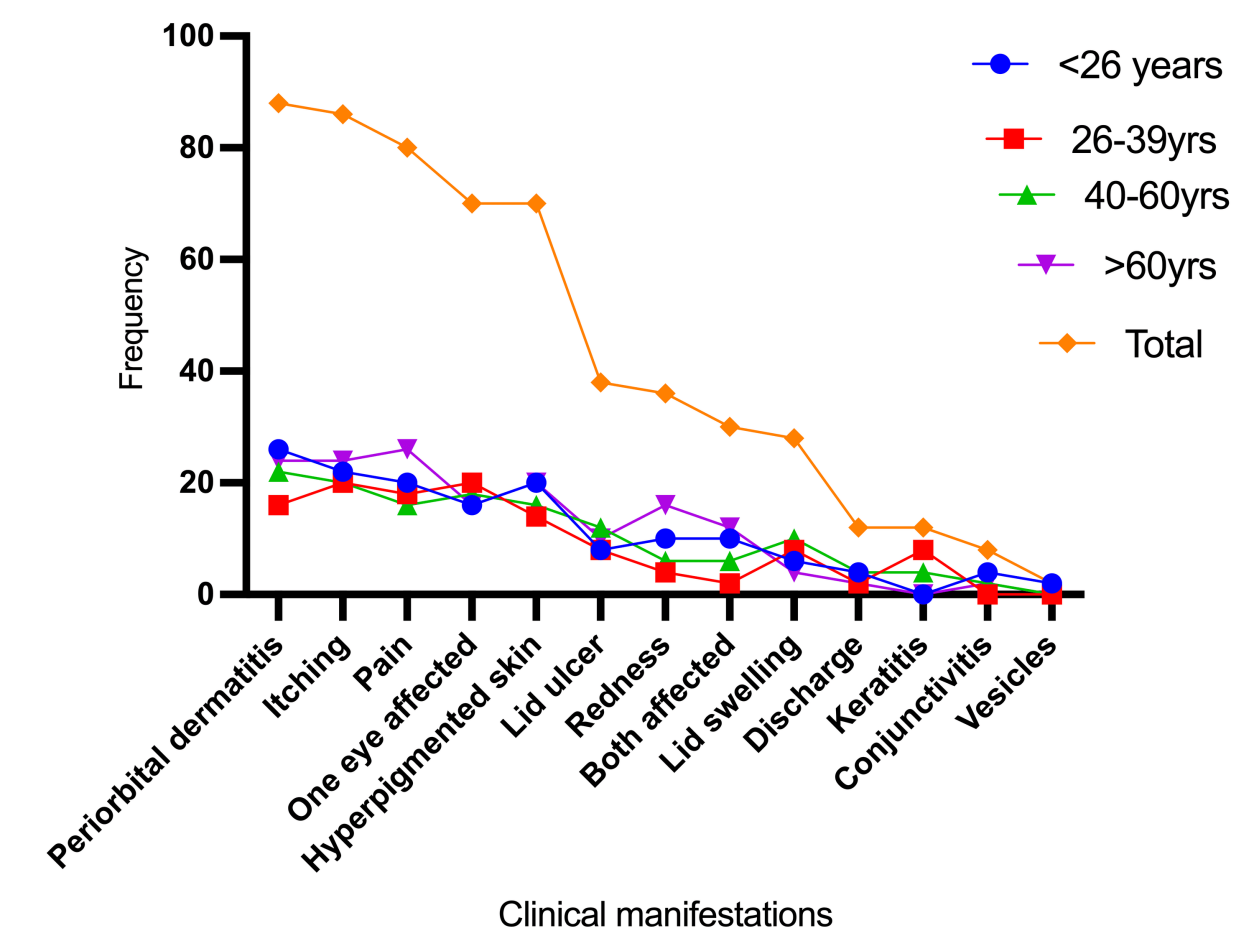
Distribution of the patients by age and clinical manifestations

**Figure 5:**
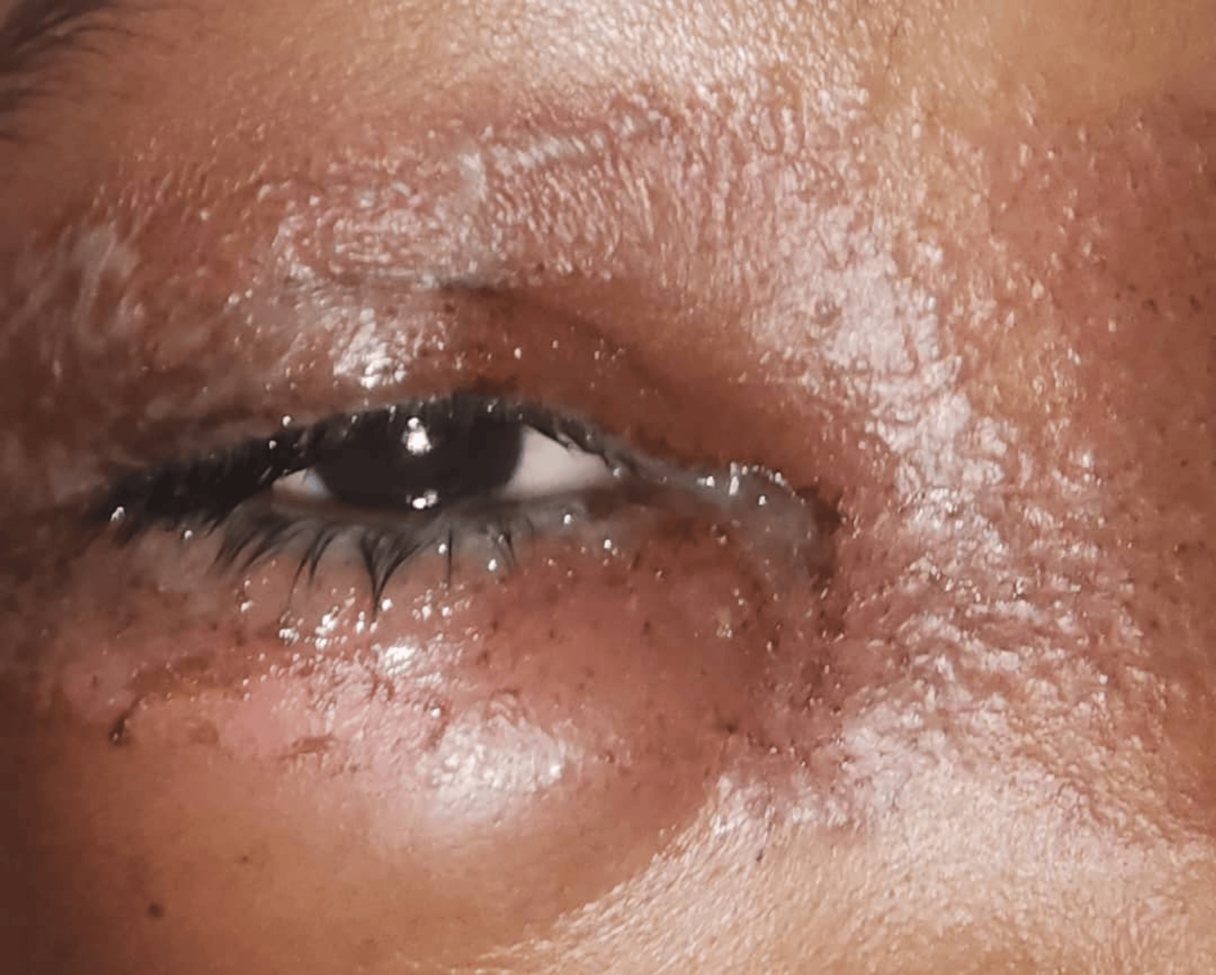
Periocular dermatitis with lid ulcer, lid swelling, vesicles and discharge

**Figure 6:**
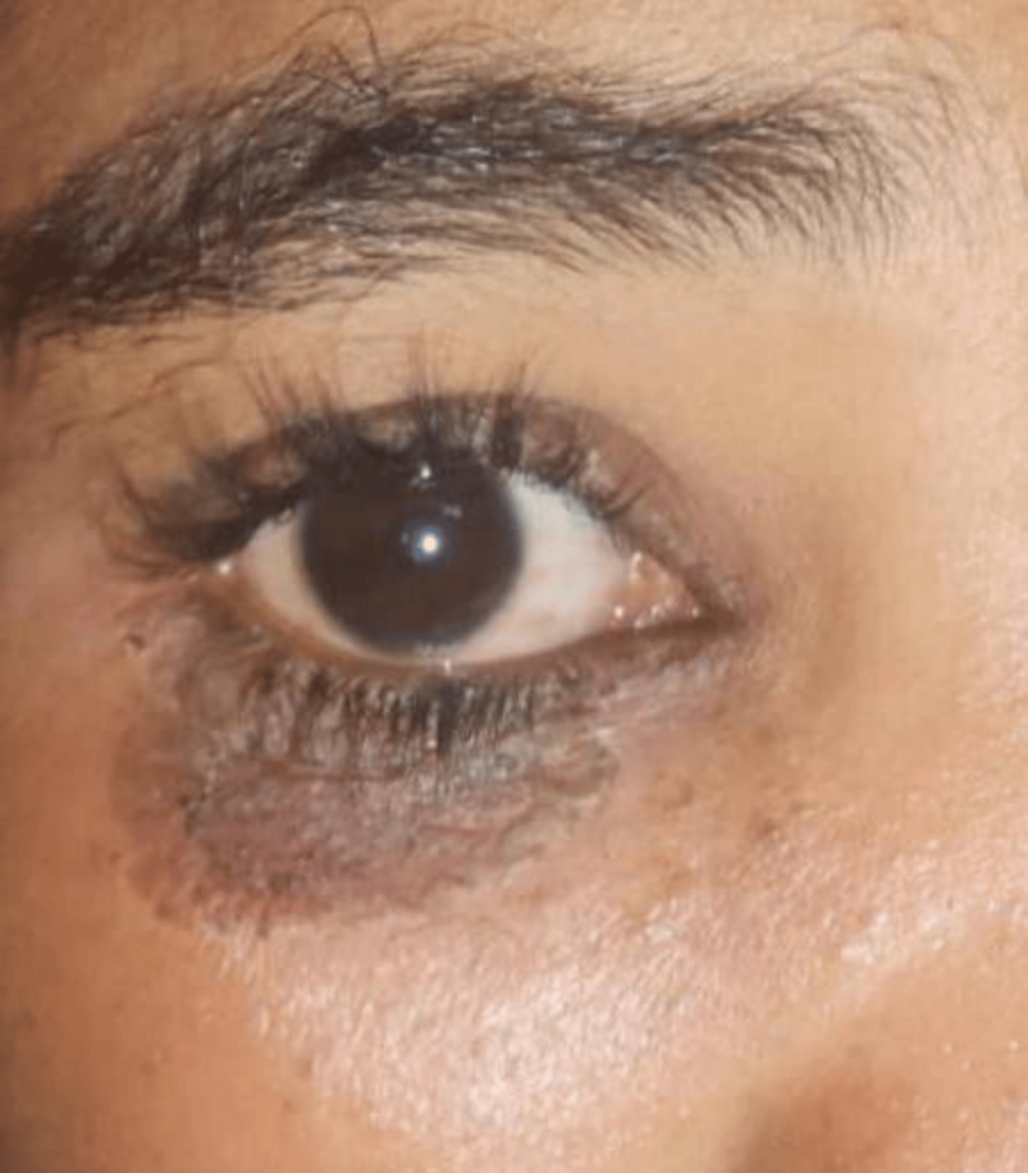
Hyperpigmented periocular skin after recovery

Table 2 and Figure 4 comprehensively summarize patient distribution by age and clinical symptoms. As seen in Figure 4 and Table 2, most clinical symptoms have similar ages. The most common symptoms are showcased in Figures 5-6.

**Table 2:** Patients categorised by age and clinical manifestations

Clinical manifestations	<26 years	26 to 39 years	40 to 60 years	>60 years	Total
Periorbital dermatitis	26	16	22	24	88
Itching	22	20	20	24	86
Pain	20	18	16	26	80
One eye affected	16	20	18	16	70
Hyperpigmented skin	20	14	16	20	70
Lid ulcer	8	8	12	10	38
Redness	10	4	6	16	36
Both affected	10	2	6	12	30
Lid swelling	6	8	10	4	28
Discharge	4	2	4	2	12
Keratitis	0	8	4	0	12
Conjunctivitis	4	0	2	2	8
Vesicles	2	0	0	0	2

## Discussion

The ongoing armed conflict in Sudan has profoundly compromised the health system, leading to the emergence of various infectious disease outbreaks. Furthermore, the rapid and elevated frequencies of population displacement have led to a significant dissemination of infectious diseases.

The cases of *Paederus*-related dermatitis have been commonly documented in the East African region, particularly in Tanzania and Uganda, with infrequent occurrences noted in South Sudan [7,11,9]. Nonetheless, to our knowledge, this study is the first report from Sudan. Since the beginning of July, there has been a significant outbreak of the *Paederus* insect, leading to an epidemic of acute dermatitis in the central western regions of the country. In the current investigation, we exclusively documented instances involving eye contact that culminated in significant ocular dermatitis.

The diagnostic challenge presented by this condition lies in its ability to closely resemble other diseases, including infectious periorbital cellulitis and herpes viral infection. Consequently, a thorough understanding of the clinical manifestations will guide us toward appropriate management of the condition and reduce the likelihood of complications [12]. We aimed to inform about this outbreak, which occurred for the first time in Sudan, and to evaluate the most prevalent clinical manifestations linked to paederin-related ocular dermatitis.

The present investigation revealed that the majority of patients exhibited periorbital dermatitis, accompanied by sensations of itching and pain. Significant intraocular signs have been previously documented as presenting features in patients with periocular *Paederus *dermatitis [13]. Ocular manifestations typically arise as a consequence of the transference of paederin via the fingers from other regions of the integument, predominantly from the exposed areas of the face, neck, or arms. Nonetheless, the ocular region may represent the sole site of involvement, as observed in all cases documented in the current article.

The ocular manifestation is typically unilateral, attributed to paederin, and presents as periorbital dermatitis, which may occur with or without keratoconjunctivitis. Individuals experiencing mild periorbital dermatitis endure persistent discomfort from the initial onset, accompanied by the emergence of slight erythema within a 24-hour period, which typically persists for around 48 hours. Instances of moderate to severe toxin exposure manifest as significant neuralgia and pronounced erythema approximately 24 hours post-contact, subsequently progressing to a vesicular stage 48 hours thereafter. The vesicles, which are not confined to a single dermatome, typically present in a linear fashion, gradually enlarging and reaching their maximal development within an additional 48 hours. Subsequently, a squamous phase ensues, during which the vesicles undergo umbilication, desiccate over a period of approximately 10 to 12 days, and subsequently exfoliate, resulting in hyperpigmented scars that may endure for a month or longer. The vesicles have the potential to evolve into pustules. The toxin exhibits characteristics of a weak base and lacks significant acid-base properties upon entering the eye, resulting in conjunctivitis, with or without accompanying keratitis. In contrast to other chemicals, this toxin lacks the ability to penetrate the cornea, resulting in inflammation confined to the deeper structures; thus, the resultant damage is restricted to the cornea and conjunctiva. The sole complication observed was post-inflammatory hyperpigmentation in the periorbital region, which resolved within one month [6,14].

The results of the current investigation indicate that ocular dermatitis associated with *Paederus *occurs more frequently in males than in females. This particular condition, *Paederus* ocular dermatitis, has the potential to impact individuals across all demographics, yet it appears to be more prevalent among males, likely attributable to their occupational or lifestyle choices, including engagement in outdoor work or the practice of sleeping with windows ajar [6]. Ophthalmologists, particularly those practicing in endemic regions, must possess a comprehensive understanding of this condition, not only for effective management but also for the purpose of educating patients regarding preventive strategies.

This study presents, for the first time, a report on the *Paederus *outbreaks in Sudan. However, it is not without its limitations. It focuses solely on ocular effects, leaving out other forms of dermatitis. Moreover, this is a single-center report, with missing laboratory identification of the toxins and a lack of follow-up.

## Conclusions

The widespread outbreak of *Paederus* dermatitis could be linked to the ongoing armed situation in Sudan. Although the ocular clinical presentation mimics that observed in other *Paederus* epidemic areas, careful evaluation is required to rule out similar disorders. To the best of our knowledge, this is the first report of a *Paederus* outbreak in Sudan. We need more research to determine the mode of transmission; *Paederus* dermatitis was previously identified in neighboring South Sudan. *Paederus* rarely comes in contact with the eye; therefore, paederin contamination of the eye warrants further investigation.
